# Response of AM fungi spore population to elevated temperature and nitrogen addition and their influence on the plant community composition and productivity

**DOI:** 10.1038/srep24749

**Published:** 2016-04-21

**Authors:** Tao Zhang, Xue Yang, Rui Guo, Jixun Guo

**Affiliations:** 1 Institute of Grassland Science, Northeast Normal University, Key Laboratory of Vegetation Ecology, Ministry of Education, Changchun 130024, China; 2 State Key Laboratory of Desert and Oasis Ecology, Xinjiang Institute of Ecology and Geography, Chinese Academy of Sciences, Urumqi 830011, China; 3 Institute of Environment and Sustainable Development in Agriculture, Chinese Academy of Agricultural Sciences, Key Laboratory of Dryland Agriculture, Ministry of Agriculture, Beijing 100081, China

## Abstract

To examine the influence of elevated temperature and nitrogen (N) addition on species composition and development of arbuscular mycorrhizal fungi (AMF) and the effect of AMF on plant community structure and aboveground productivity, we conducted a 5-year field experiment in a temperate meadow in northeast China and a subsequent greenhouse experiment. In the field experiment, N addition reduced spore population diversity and richness of AMF and suppressed the spore density and the hyphal length density (HLD). Elevated temperature decreased spore density and diameter and increased the HLD, but did not affect AMF spore population composition. In the greenhouse experiment, AMF altered plant community composition and increased total aboveground biomass in both elevated temperature and N addition treatments; additionally, AMF also increased the relative abundance and aboveground biomass of the grasses *Leymus chinensis* (Poaceae) and *Setaria viridis* (Gramineae) and significantly reduced the relative abundance and aboveground biomass of the *Suaeda corniculata* (Chenopodiaceae). Although elevated temperature and N addition can affect species composition or suppress the development of AMF, AMF are likely to play a vital role in increasing plant diversity and productivity. Notably, AMF might reduce the threat of climate change induced degradation of temperate meadow ecosystems.

Arbuscular mycorrhizal fungi (AMF) play a vital role in the survival and development of plants in terrestrial ecosystems by improving growth and increasing productivity^1^,^2^,^3^, which determines ecosystem stability and multifunctionality^4^,^5^,^6^. Moreover, AMF influence many other ecosystem processes^7^, including carbon (C) cycling by accelerating the decomposition of organic matter^7^,^8^,^9^ and nitrogen (N) cycling by improving the uptake and transfer of nitrogen^10^,^11^ and by reducing nitrogen leach^12^, among other processes. However, the species composition and the development of AMF are also influenced by changes in climate, such as elevated temperature and nitrogen addition, although the ecological determinants that regulate these parameters of AMF are not well understood.

Global climate change induced by human activities influences the development and species composition of AMF. Elevated temperature increases the allocation of carbon to AMF and hyphae length^13^,^14^ and improves mycorrhizal colonization of root systems^15^,^16^. However, in several studies, elevated temperature had no effect on the length of roots colonized, spore density or the extraradical hyphal density of AMF^13^,^17^. Elevated temperature not only influences the development of AMF in soil and roots but also alters the species composition. The relative abundance and function of AMF are influenced by the variation of seasons and temperatures^18^, and the effect of elevated temperature on the species richness of AMF in soil can be positive^19^ or have no effect on AMF species composition^17^. These observations of varying responses suggest that the effects of elevated temperature on AMF are not uniform. For instance, warming increased AMF species richness in a semiarid steppe ecosystem^19^ in northern China, while warming did not affect AMF species composition in a native grassland in UK^17^ and Qinghai Tibet Plateau in China^20^.

As one of the major threats to ecosystem stability, nitrogen (N) deposition reduces plant species number^21^. Moreover, N inputs also have an important effect on species composition in the AMF community^22^. N fertilization significantly decreased AMF spore abundance, but no impact on spore species richness and AM hyphal density in a field experiment at the Cedar Creek Ecosystem Science Reserve, MN, USA^23^. The result from an alpine meadow ecosystem of China found that N fertilization reduced the abundance of Glomeromycota and AMF species richness^24^. In many studies, a reduction in the length of root colonization has been observed^23^,^24^, but other studies have reported an increase in the AMF colonization of roots after N fertilization^25^. Although the separate effects of elevated temperature and N addition on the AM fungal community have received considerable attention^15^,^23^,^24^, global elevated temperature and N deposition frequently occur simultaneously. Elevated temperature frequently enhances soil microbial activity and increase N mineralization^26^. Although limited, the effects of combined elevated temperature and nitrogen fertilization on soil microorganisms have been examined^27^,^28^,^29^. Nevertheless, the integrated effects of elevated temperature and N addition on AM fungal communities in temperate meadow ecosystems in China are not well understood.

Many studies demonstrated that plant-induced alterations of the soil affect the structure of the plant community^30^,^31^,^32^. Negative plant-soil feedback plays an important role in maintaining plant community diversity^33^,^34^,^35^, whereas positive plant-soil feedback may reduce plant diversity^36^,^37^. However, it is unclear which type of plant-soil feedback will occur during climate change.

The Songnen grassland is located at the eastern edge of the Eurasian grassland and is the largest and most typical meadow steppe in China^38^. The average temperature of the Songnen meadow steppe has increased by 2 °C in the last twenty years^39^. The average atmospheric N deposition is approximately 10.5 g m^−2^ yr^−1^.^40^ Therefore, to study the effects of elevated temperature and N addition on plant community composition and productivity, a field experiment was established in 2006 in the Songnen meadow ecosystem^41^. However, the responses of AMF to elevated temperature and N addition and the effects of changes in AMF on the plant community composition and productivity are not clear in the Songnen meadow ecosystem in northeastern China.

To better understand the effects of elevated temperature and N addition on the development and species community composition of AMF communities and their feedbacks on the aboveground plant community, a 5-year elevated temperature and N addition field experiment and a greenhouse experiment were conducted. We hypothesized that (1) N addition and elevated temperature would affect the development and species composition of AMF in the field experiment and that (2) AMF would alter the composition of the plant community and increase plant diversity and productivity under the elevated temperature and N addition conditions in the greenhouse experiment because of AMF can reduce the negative of N addition and elevated temperature on plant growth.

## Results

### Spore development and species composition of AMF in the field experiment

The elevated temperature treatment decreased the AMF spore density and diameter by 32% (*P* < 0.05) and 10% (*P* < 0.05), respectively, and increased the hyphal length density (HLD) by 27% (*P* < 0.001). The N addition treatment decreased the AMF HLD 14% (*P* < 0.05, Table 1). Significant interactive effects of elevated temperature × N addition on AMF spore density and diameter were detected (all *P* < 0.01), but no interactive effect on HLD was observed (*P* > 0.05; Table 1).

The species composition of AMF community was significantly affected by the elevated temperature and N addition treatments. Elevated temperature had no impact on AMF species richness, diversity (*H*) or evenness (*E*) (all *P* > 0.05; Table 1), whereas N addition decreased AMF species richness and *H* by 18% (*P* < 0.05) and 25% (*P* < 0.05), respectively, compared with control, but had no impact on *E* (Table 1). No interactive effects of elevated temperature × N addition on *H* and *E* were detected (all *P* > 0.05), but a significant interactive effect of elevated temperature × N addition on species richness was observed (*P* < 0.05).

### Plant community composition in the greenhouse experiment

**Figure 1:**
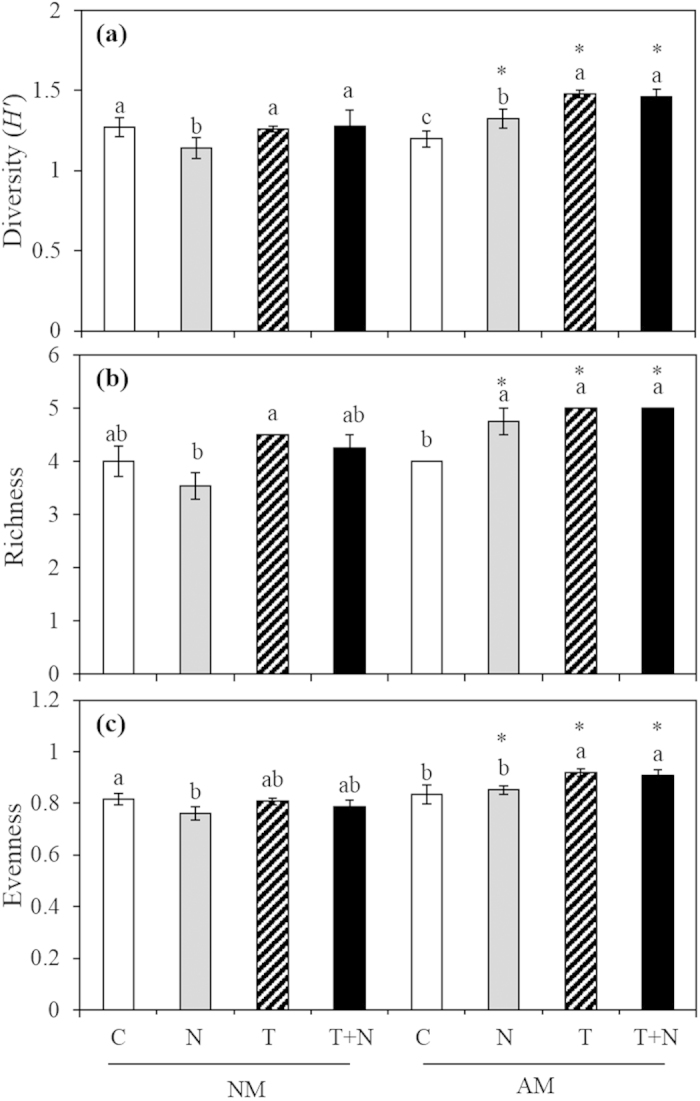
Effects of AMF on plant diversity (**a**), richness (**b**) and evenness (**c**) under elevated temperature and N addition in the greenhouse experiment. The lowercase letters in each column represent significant difference (*P* < 0.05) among different treatments in the same AMF treatment. Asterisks represent significant difference (*P* < 0.05) between with and without AMF treatments in the same N, T or T + N treatments.

AMF significantly increased plant diversity (Fig. 1a), richness (Fig. 1b) and evenness (Fig. 1c) both in elevated temperature and N addition treatments. In elevated temperature treatment, AMF significantly increased plant species diversity, richness and evenness, the mycorrhizal benefits of them were 17.1, 11.1 and 13.6, respectively (all *P* > 0.05). In N addition treatment, AMF significantly altered plant community composition, the mycorrhizal benefits of plant diversity, richness and evenness were 16.3 33.8 and 11.7, respectively, and significant differences between AM and NM were observed (all *P* > 0.05). Compared to control, the mycorrhizal benefits on plant diversity, richness and evenness all significantly increased (all *P* < 0.05) both in elevated temperature and N addition treatments. Significant interactive effects of N addition × AMF on plant diversity, richness and evenness were observed, and significant interactive of elevated temperature × AMF on plant diversity and evenness were also observed. No interactive effects of elevated temperature × N addition × AMF on plant species diversity, richness and evenness were detected (Table 2).

**Figure 2:**
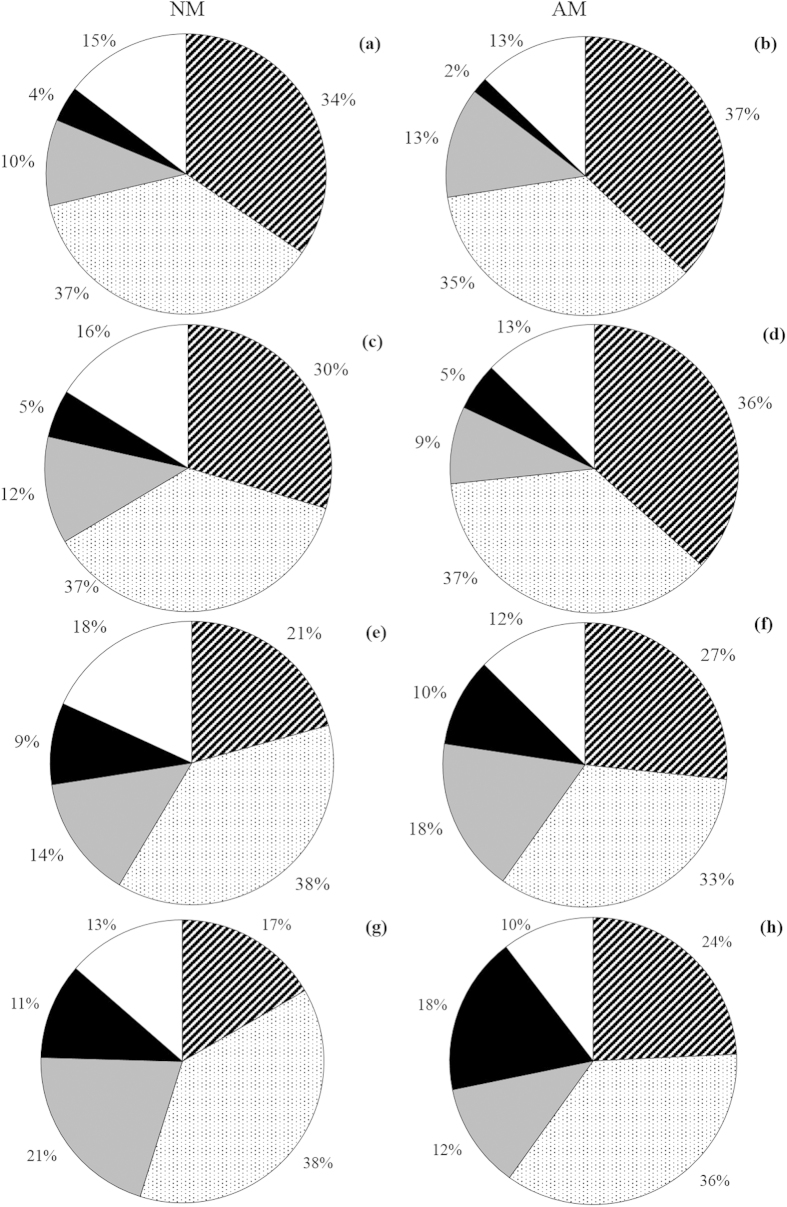
Species density composition (%) of total number of individuals in four control (**a,b**), N addition (**c,d**), elevated temperature (**e,f**), and elevated temperature plus N addition treatments (**g,h**) with AMF (AM) or without AMF (NM) in the greenhouse experiment. Species included are (clockwise from top) *L. chinensis* (oblique line); *S. viridis* (dots); *L. davurica* (grey); *C. virgata* (black); *S. corniculata* (clear). Data represent the mean of six replicates.

AMF altered plant species composition both in elevated temperature and N addition treatments (Fig. 2). In elevated temperature, AMF significantly increased the abundance of *L. chinensis*, *L. davurica* and *C. virgata*, the mycorrhizal benefits of them were 32.5, 29.6 and 11.1 (all *P* < 0.01), respectively; but the abundance of *S. viridis* and *S. corniculata* significantly decreased by AMF and the mycorrhizal benefits were −9.6 (*P* < 0.05) and −28.6 (*P* < 0.05). In N addition treatment, AMF had no impact on the abundance of *S. viridis* (*P* > 0.05) and *C. virgata* (*P* > 0.05), and AMF decreased abundance of *S. corniculata* (*P* < 0.05) and the mycorrhizal benefit was −20.3 whereas AMF increased the abundance of *L. chinensis* and *L. davurica* and the mycorrhizal benefits of them were 25.0 (*P* < 0.05) and 16.7 (*P* < 0.05). Significant interactive effects of N addition × AMF on the abundances of *L. chinensis* and *S. corniculata* were observed, no significant interactive effects of elevated temperature × N addition, elevated temperature × AMF, and elevated temperature × N addition × AMF were detected (Table 3).

### Aboveground plant biomass in the greenhouse experiment

**Figure 3:**
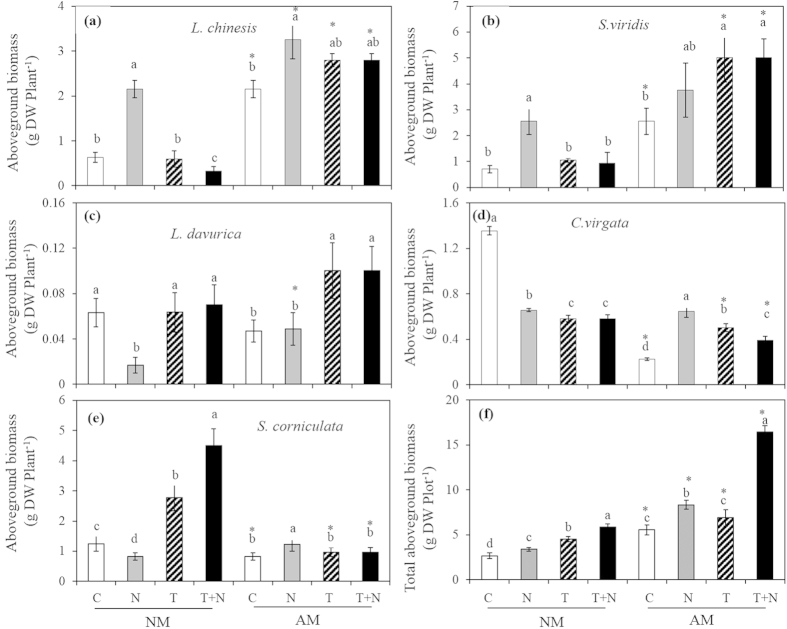
The effects of AMF on plant biomass under N addition and elevated temperature in the greenhouse experiment. (**a**) *L. chinensis*; (**b**) *S. viridis*; (**c**) *L. davurica*; (**d**) *C. virgata*; (**e**) *S. corniculata*; (**f**) Total aboveground biomass. The lowercase letters in each column represent significant difference (*P* < 0.05) among different treatments in the same AMF treatment. Asterisks represent significant difference (*P* < 0.05) between with and without AMF treatments in the same N, T or T + N treatments.

In control, AMF increased aboveground biomass of *L. chinensis* and *S. viridis* (Fig. 3), and the mycorrhizal benefits were 240.6 (*P* < 0.01) and 258.5 (*P* < 0.01), respectively, but significantly decreased the biomass of *C. virgat*a (*P* < 0.05) and *S. corniculata* (*P* < 0.05). In elevated temperature treatment, aboveground biomass of *L. chinensis*, *S. viridis*, *L. davurica* significantly increased by AMF, the mycorrhizal benefits of them were 366.0 (*P* < 0.01), 374.4 (*P* < 0.01) and 57.9 (*P* < 0.05), respectively, AMF decreased aboveground biomass of *C. virgata* and *S. corniculata* significantly. In the N addition treatment, AMF increased aboveground biomass of *L. chinensis*, *S. viridis* and *L. davurica*, the mycorrhizal benefits were 51.1, 47.4 and 186.8, respectively, whereas the aboveground biomass of *S. corniculata* decreased significantly. No significant interactive effects of elevated temperature × N addition, N addition × AMF, or elevated temperature × N addition × AMF on the aboveground biomass of *L. davurica* and *C. virgata* were detected (Table 3). Significant main effects of elevated temperature, N addition, AMF and interactive effects of elevated temperature × N addition and elevated temperature × N addition × AMF on the aboveground biomass of *L. chinensis* and *S. corniculata* were detected (Table 3). Moreover, AMF significantly increased total aboveground biomass both in elevated temperature and N addition treatments (Fig. 3f).

### Plant biomass composition in the greenhouse experiment

**Figure 4:**
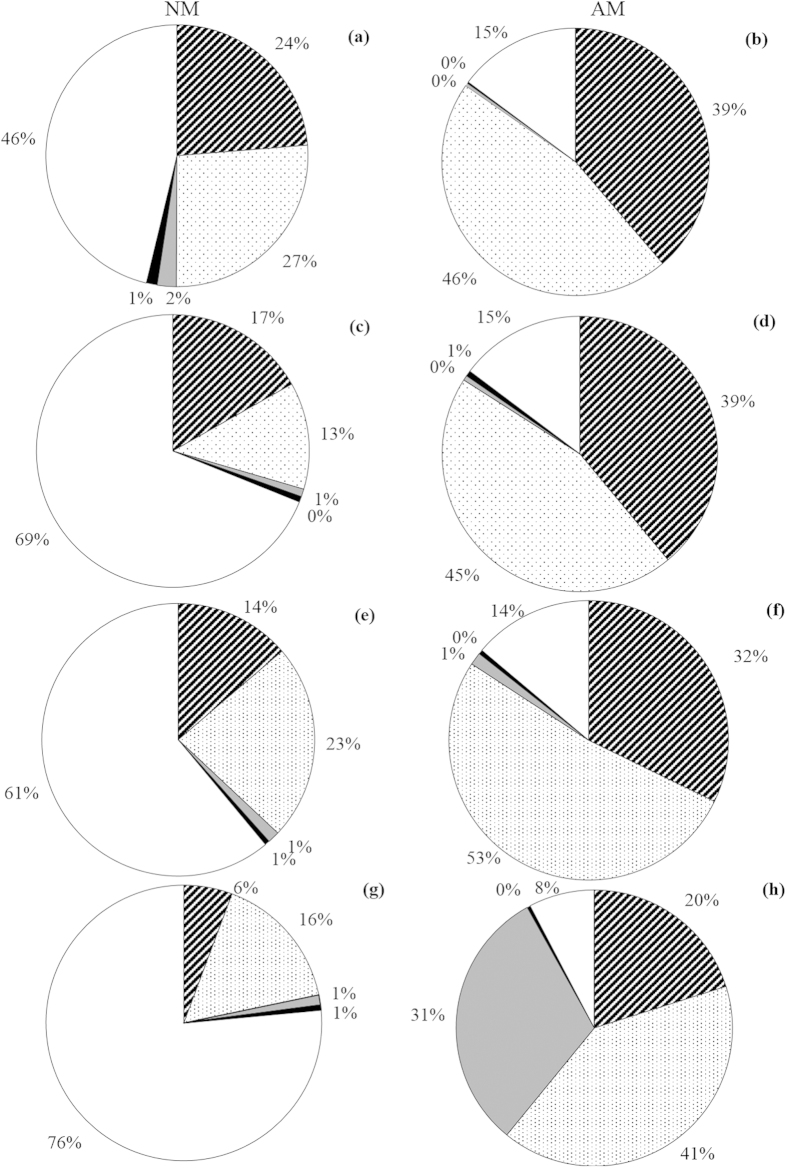
Species composition (%) of total aboveground biomass in four control (**a,b**), N addition (**c,d**), elevated temperature (**e,f**), and elevated temperature plus N addition treatments (**g,h**) in pots with AMF (AM) or without AMF (NM). Species included are (clockwise from top) *L. chinensis* (oblique line); *S. viridis* (dots); *L. davurica* (grey); *C. virgata* (black); *S. corniculata* (clear). Data represent the mean of six replicates.

AMF altered the relative contribution of each plant species to the total aboveground biomass in elevated temperature and N addition treatments (Fig. 4). In elevated temperature treatment, AMF significantly increased the proportion of total plant from *L. chinensis* and *S. viridis*, the mycorrhizal benefits were 135.4 (*P* < 0.01) and 124.8 (*P* < 0.01), respectively, whereas decreased the proportion of total plant from *C. virgata* and *S. corniculata* and the mycorrhizal benefits were −13.7 (*P* < 0.05) and −77.2 (*P* < 0.05), respectively. Compared to control, the mycorrhizal benefits of *L. chinensis*, *S. viridis* and *L. davurica* significantly increased, but the mycorrhizal benefits of *S. corniculata* decreased. In the N addition treatment, AMF also increased the proportion of total plant from *L. chinensis* and *S. viridis*, the mycorrhizal benefits were 133.7 (*P* < 0.01) and 259.5 (*P* < 0.01), whereas decreased the proportion of total plant from *L. davurica* and *S. corniculata* and the mycorrhizal benefits were −35.7 (*P* < 0.05) and −78.4 (*P* < 0.05), respectively. The mycorrhizal benefits of *L. chinensis*, *S. viridis* and *L. davurica* in elevated temperature and N addition treatments significantly increased (all *P* < 0.05) compared to control, but the mycorrhizal benefits of *S. corniculata* decreased, significantly. Additionally, significant main effects of AMF and interactive effects of AMF × N addition on the proportions of *L. chinensis* and *S. corniculata* to total biomass were observed (Table 3).

## Discussion

Based on our results, changes in climate (elevated temperature and N addition) affected the development of AMF and determined the composition of AMF spores population. Although the effect of elevated temperature on the composition of the AMF has been examined in many field studies of different ecosystems^20^,^28^,^42^, the response of the AMF was not consistent in these studies. In the temperate steppe (Inner Mongolia, China), elevated temperature significantly affected the relative spore abundance of AMF species and decreases AMF diversity^42^, whereas elevated temperature has no significant effect on the composition of the AMF community in an alpine meadow ecosystem in China^20^ and a native grassland in the UK^17^. These inconsistent results may indicate different responses of AMF communities in different ecosystems to elevated temperature because the AMF species composition often is determined by environment and plant community composition^23^,^44^. In the current study, although elevated temperature significantly altered the development of AMF (Table 1), elevated temperature did not affect AMF spore population diversity and evenness; this result is consistent with the absence of a significant effect of elevated temperature on the species composition of the plant community^41^. However, an interactive effect of elevated temperature × N addition on AMF species richness was observed, which suggesting that the effects of elevated temperature on AMF species richness might be influenced by soil N availability. In the present study, elevated temperature decreased AMF spore density and diameter but increased the HLD significantly, which is consistent with a result from a simulated greenhouse experiment^15^, but not with the result from a field experiment in the Qinghai-Tibetan Plateau^20^. The distinction between our result and other results suggest that the influence of elevated temperature on AMF might be related to the climatic conditions of experiment ecosystem, such as, temperature. Our HLD results indicate that plants might have higher tolerance to high temperatures because an increase in HLD would increase the absorption of water and nutrients.

In the current study, N addition significantly decreased the species diversity and richness of the AMF spore population. These findings are consistent with earlier studies that show that N fertilization decreases the spore richness and abundance of AMF^23^,^24^,^43^. Moreover, N addition significantly reduced the biomass, spore density and diameter, and HLD of AMF significantly, which is consistent with earlier results from Cedar Creek^23^,^44^, an alpine meadow ecosystem^24^. Based on the current results, the community composition and the development of AMF would respond quickly to N addition in the N-limited Songnen meadow ecosystem. In many studies, plant community composition determines the spore richness and biomass of AMF^23^,^24^,^44^. Therefore, the reduction of the richness and biomass of AMF in this ecosystem might be related to changes in the composition of plant community induced by N addition, given that our earlier study showed that N addition significantly increased the percentage in term of both number of plants and biomass of mycorrhizal species and reduced the percentage of non and poorly mycorrhizal species^41^.

In our study, significant interactive effects of elevated temperature × N addition on AMF spore density and diameter and species richness were observed. However, we did not detect significant interactive effects of elevated temperature × N addition on plant diversity, richness or aboveground productivity^41^. Thus, the factors that determine the species composition and development of AMF are likely different from those of plant communities.

A majority of the previous field and greenhouse studies demonstrate that AMF determine the structure and productivity of the plant community^4^,^5^,^45^,^46^. However, the species composition and the development of AMF are significantly influenced by global changes that include the following: N deposition^23^,^24^,^44^, warming^41^,^42^, and elevated CO_2_^15^,^23^. Thus, changes in the species composition or in the development of AMF results in plant-soil feedbacks that regulate plant community structure and productivity^36^,^47^. However, the effects of plant-soil feedbacks on the plant community during climate change are not well understood. In the current study, although N addition significantly decreased the species richness and diversity of AMF spore population in the field experiment (Table 1), AMF significantly increased plant species diversity and richness via increasing the competition of non nitrophilous species (*L. chinensis* and *S. viridis*) with the addition of N in the greenhouse experiment (Fig. 1). These results are consistent with a previous study in which AMF reduced the negative effects of nitrogen enrichment on plant community structure in a dune grassland ecosystem^48^. The present results suggest that AMF might help improve the adaptability of plant species to N deposition and help maintain plant diversity in temperate meadow ecosystems due to AMF improved plant growth and increased plant biomass (Fig. 3). Furthermore, although elevated temperature had no impact on AMF species composition, AMF significantly increased plant species diversity and richness in the elevated temperature treatment condition (Fig. 1). Our results suggest that AMF improves growth of plants with high mycorrhizal benefits and tolerance to elevated temperatures, but further investigation is required to determine the mechanisms controlling the influence of AMF on the development of plant species under elevated temperature conditions.

AMF altered the relative abundance of plant species (%) in the elevated temperature and N addition treatments in the greenhouse experiment. The relative abundance of *L. chinensis* significantly increased in the elevated temperature, N addition, and elevated temperature plus N addition treatments, but the relative abundance of *S. corniculata* decreased (Fig. 2) with AMF. This result might be related to the level of mycorrhizal dependence of different plant species; for example, AMF improved the seedling establishment of the mycorrhizal *L. chinensis* in the elevated temperature and N addition treatments but had no impact on the nonmycorrhizal plant *S. corniculata*. These results are consistent with the earlier findings that AMF increased the abundance and biomass of highly mycorrhizal species and reduced the abundance of nonmycorrhizal plants^45^,^49^. Moreover, the effects of AMF on plant community structure are affected by the species composition of the AMF community^50^,^51^,^52^. Therefore, under conditions of climate change, the feedbacks between the species of AMF and plant species should be considered in future studies. The species *S. corniculata* is a typical indication of grassland degradation caused by grazing and mowing in the Songnen meadow steppe, and with the aggravation of degradation the number of *S. corniculata* highly increase^53^. Based on our results, AMF might reduce the threat of grassland degradation caused by climate change or overgrazing.

A large number of studies have demonstrated that AMF can improve aboveground productivity^1^,^54^,^55^. In the greenhouse experiment, AMF significantly increased total aboveground biomass which is consistent with the previous studies^1^,^3^,^54^. This result suggests that AMF still play a vital role in determining ecosystem net primary productivity under climate change of elevated temperature and N addition, which would affect carbon sequestration and influence C and N cycling in grassland ecosystem. In an earlier study, AMF were found to influence C and N cycling by altering the plant C:N:P stoichiometry^56^. However, the influence of AMF on plant C:N stoichiometry under elevated temperature and N addition conditions requires further study. The biomass of the plant community was also altered under the elevated temperature and N addition treatments by AMF. In both the elevated temperature and the N addition treatments, the proportion of biomass from *L. chinensis* and *S. viridis* increased significantly with AMF, whereas the proportion of biomass from *S. corniculata* decreased significantly. These results are consistent with early findings in a semiarid herbland in South Australia^45^. The changes in the composition of plant community biomass caused by AMF might be determined by the different mycorrhizal responses of plant species. In the current study, significant differences in mycorrhizal response among the species were observed (Fig. 3), positive mycorrhizal benefits of *L. chinensis*, *S. viridis* and *L. davurica* and negative mycorrhizal benefits of *C. virgata* and *S. corniculata* were detected. In the Songnen grassland, the species in the grass family (e.g., *L. chinensis* and *S. viridis*) are the most important forages, whereas the species in Chenopodiaceae family (e.g., *S. corniculata*) are weeds that livestock do not like to eat. Therefore, these results suggest that AMF can significantly improve the productivity of forage and reduce the productivity of harmful weeds, which would sustain livestock production. Furthermore, the plant species *S. corniculata* is a typical indicator of degradation in the Songnen grassland, and our results indicate that that AMF can reduce the risk of grassland degradation under conditions of climate change by suppressing the growth of *S. corniculata*.

## Conclusions

Elevated temperature and N addition treatments, proxies for global climate change, altered the spore population composition and suppressed the development of AMF. However, AMF reduced the negative effects of elevated temperature and N addition on plant diversity and productivity and increased the contribution of grasses thereby increasing the productivity of grassland. Thus, AMF have the potential to reduce the degradation of grasslands caused by elevated temperature and N addition.

## Methods

### Study site

The experiment was conducted at the Songnen Grassland Ecological Research Station (44°45′ N, 123°45′ E), Northeast Normal University, Jilin Province, in northeast China. The grassland is situated at the eastern edge of the Eurasian steppe and is characterized as Eurasian continental meadow steppe. Mean annual precipitation is approximately 400 mm, 90% of which occurs from May to October. Annual average air temperature is 4.9 °C, and annual average land surface temperature is 6.2 °C. The soil in the study area is a soda-saline soil, and the soil pH was 8.2, with 3–4% organic matter in the soil surface layer. The vegetation of the experimental site is dominated primarily by *Leymus chinensis* (Trin. Tzvel, Poaceae)*; Chloris virgata* (Sw, Gramineae)*, Setaria viridis* (L., Gramineae), *Lespedeza davurica* (Laxm., Leguminosae) and *Suaeda corniculata* (C. A. Mey, Chenopodiaceae) are the main companion species.

## Field experiment: Effects of elevated temperature and N addition on the development and species composition of AMF

### Experimental design

The experiment was a completely randomized block factorial experiment with two factors: elevated temperature and N addition. There were four treatments: control (C), elevated temperature (T), N addition (N), and elevated temperature plus N addition (T + N), with 6 replicates each. The size of the plots was 2 m × 3 m. The plots in the elevated temperature treatment were all heated continuously by infrared radiators (MSR-2420, Kalglo Electronics Inc., Bethlehem, PA, USA) that were suspended 2.25 m over the plot center. In each control or N addition plot, one ‘dummy’ heater of the identical shape and size was installed to mimic the shading effects of the infrared radiator. The heaters in the elevated temperature treatment were set for approximately 2000 W of radiation output. In northern, temperate grassland ecosystems, the saturation rate for N deposition is approximately 10 g m^−2^ yr^−1^^39^. Therefore, in the N addition treatment; ammonium-nitrate (10 g m^−2^ yr^−1^) was added as an aqueous pulse on the first day of May each year. In the control and elevated temperature plots, an identical amount of water (without N) was added to account for that added in N addition condition. The experiment began in May 2006 and was terminated in September 2010.

### Soil sampling

Three soil cores (15 cm in depth, 5 cm in diameter) were randomly collected and were mixed to create a composite sample at the end of the experiment. Fresh soil samples were sieved (2-mm) to remove roots and debris for AMF spore isolation.

### Extraradical hyphae

The extraradical hyphae from the homogenized soils were extracted and stained with trypan blue using the method of Jakobsen *et al.*^57^. The hyphal length density (HLD) was quantified using a compound microscope with a gridded reticule at 250x magnification.

### Spore separation and identification

The spores were extracted using wet sieving and sucrose centrifugation^58^, mounted on glass slides with alcohol lacto-glycerol (PVLG) and were examined under 100–1000x magnification with a microscope. The species of each spore was identified (Table S1) using taxonomic criteria^59^, the information published by INVAM (http://invam.caf.wvu.edu/) and Schüβler’s Glomeromycota Species List (http://www.lrz.de/~schuessler/amphylo/). The slides are maintained at Northeastern Normal University. A sporocarp was counted as a single spore. The spore density is the number of AM fungal spores in 20 g of soil. The average spore diameter was calculateed for each soil sample.

The species richness (SR) = number of AM fungal taxa found in 20 g of soil and the relative abundance (RA) = (number of spores of a species or genus/total spores) × 100. The number of spore population and the density were also used to calculate species richness (mean number of AMF species per 20 g of soil), diversity (Shannon-Weiner *H′*) and evenness.

## Greenhouse experiment: The feedbacks of AMF on plant community composition and productivity

### Soil and plants

The soil used in this experiment was collected from the grassland experimental sites. The soil was sterilized 2 times using high-pressure steam for 2 h at 121 °C per time. When the first sterilization completed, the soil were homogenized and then sterilized again. To isolate the AMF spores, we collected the soil (500 g) from the field plots under treatment for 5 years with elevated temperature and N addition. The isolated AMF spores were cultured using *Medicago sativa* with 200 g of sterilized soil. Plants were grown in phytotrons for 4 months with the temperature of 25 °C from 06:00–20:00, 18 °C from 20:00–06:00, and received deionized water to maintain soil moisture at 10–20% by weight. The entire cultured AMF from field experiment (including soil, root segments, AMF spores and hypha) was homogenized and used to inoculate the soils with AMF in the greenhouse experiment. The seeds of one dominant species *L. chinensis* and four companion species *C. virgata, S. viridis*, *S. corniculata* and *L. davurica* were collected from the Songnen meadow steppe and were stored in a refrigerator at <4 °C before use.

### Experimental design

In the greenhouse experiment, the four treatments were identical to the treatments in field experiment, with control, elevated temperature, N addition, and elevated temperature plus N addition. Each treatment was also replicated 6 times. Soil samples were collected from each treatment in the field experiment and were homogenized to use as AMF inocula in the greenhouse experiment. The sterilized soil was placed into pots, and 200 g of AMF inoculum (approx. 6000 spores) from the similar treatment (from the field experiment) was added to the soils in the AMF treatment (AM). An identical amount of the inoculum mix was sterilized using 10 k Gy ^60^Co γ-rays and was used for the non-AMF (NM) treatment. To minimize differences in the rhizosphere microbial communities of AMF and non-AMF treatments, 10 ml filtrates free from mycorrhizal propagules from the inoculum were added to the non-AMF treatments and 10 ml deionized water were added to AMF treatments. The pots were 34 cm (in diameter) × 23 cm (in depth) and filled with 2.5 kg (dry weight) soil. The seeds of the five species were surface disinfected in 10% (v/v) hydrogen peroxide for 5 min, rinsed five times with deionized water, and germinated at 20 °C. After 48 h, the germinated seeds were added to each pot, and the density of these five species was thinned based on their densities in the field and was kept consistent for all treatments. The densities of these species were *L. chinensis* 15 plants per pot, *S. viridis* 10 plants per pot, *C. virgata* 7 plants per pot, *S. corniculata* 5 plants per pot and *L. davurica* 5 plants per pot.

The experimental pots were placed in phytotrons (LT/ACR-2002, E-Sheng Tech., Beijing, China) at Northeast Normal University. In the phytotrons, light intensity was 350 μ mol^−2^ S^−1^ (06:00–20:00) daily. The relative humidity was 40–60%. In the control and N addition treatments, the temperature settings were 22 °C from 06:00–10:00, 25 °C from 10:00–15:00, 22 °C from 15:00–20:00, and 22 °C from 20:00–06:00. In the elevated temperature and elevated temperature plus N addition treatments, the temperature in each time period was elevated 3 °C compared with the control and N addition treatments. The air temperature in the phytotrons was adjusted and monitored every 10 seconds, and the temperature matched the mean summer temperature from 2000–2011 in the Songnen meadow grassland^60^. Ammonium nitrate (10 g m^−2^ yr^−1^) was added to the pots of N addition and elevated temperature plus N addition treatments. The pots were irrigated every 2 days; the soil water content was 50–60% of field capacity.

The plant species number and density of each species was observed and recorded daily at 14:00. The plants were harvested after twelve weeks of growth. The shoots and roots were removed from the pot and were washed with deionized water. The roots were cut into 1-cm segments and thoroughly mixed. A sub-sample of 0.5 g was cleared with 10% (w/v) KOH at 90 °C for 2 h and then stained with trypan blue. Mycorrhizal colonization (Table S2) was estimated according to a previously described method^61^. The shoots and remaining roots were dried at 60 °C for 48 h and then weighed to calculate plant productivity. The number and density of plant species were used to calculate the species diversity (Shannon-Weiner index *H*), richness and evenness. The mycorrhizal benefit was calculated according to the following formula. The mycorrhizal benefit = [(AM/NM) − 1] × 100.

### Statistical analysis

The statistical analyses were conducted using the SPSS statistical software package 7 (SPSS 16.0 for Windows, Chicago, IL, USA). The spore density counts and estimates of species richness were square root transformed. Other data were analyzed without transformation. Spore density and AM fungal diversity index results are shown as arithmetic mean values with standard errors. A two-way ANOVA was used to test the effects of elevated temperature, N addition and their interaction with the AM fungal HLD, spore density, species richness, diversity, and evenness in the field experiment, and a three-way ANOVA was used to test the feedback of AMF on the community composition and productivity of the plant community in the greenhouse experiment. Treatment means were compared by Tukey at *P* = 0.05. The data were all tested for normality and homogeneity of variance before analysis.

## Additional Information

**How to cite this article**: Zhang, T. *et al.* Response of AM fungi spore population to elevated temperature and nitrogen addition and their influence on the plant community composition and productivity. *Sci. Rep.* **6**, 24749; doi: 10.1038/srep24749 (2016).

## Supplementary Material

Supplementary Information

## Figures and Tables

**Table 1:** The effects of elevated temperature (T) and N addition (N) on arbuscular mycorrhizal fungi (AMF) development and species composition.

Treatments	AMF spore density (No. 20 g soil^−1)^	AMF spore diameter (μm)	AMF HLD (m g^−1^ soil)	AMF species diversity (*H*)	AMF Species evenness (*E*)	AMF species richness
C	365 ± 26a	108 ± 3a	15.3 ± 0.6b	1.93 ± 0.08a	0.74 ± 0.03a	13.67 ± 0.95a
N	178 ± 18d	55 ± 5c	13.2 ± 1.1c	1.58 ± 0.09b	0.67 ± 0.03a	10.33 ± 1.02b
T	248 ± 23c	97 ± 6b	19.5 ± 1.6a	1.99 ± 0.14a	0.74 ± 0.05a	15.00 ± 1.48a
T + N	303 ± 14b	92 ± 8b	19.4 ± 0.6a	1.86 ± 0.08a	0.71 ± 0.04a	12.00 ± 1.29a
Analysis of Variance (*F*-Values)						
T	3.3*	4.7*	25.3***	3.4NS	0.6NS	3.2NS
N	9.9**	23.9***	4.8*	4.7*	1.1NS	5.1*
T*N	33.1***	16.2**	0.02NS	2.6NS	3.3NS	4.8*

Note, the lowercase letters in each column represent significant difference among different treatments at 0.05 level. *Significant difference at *P* < 0.05; **significant difference at *P* < 0.01; ***significant difference at *P* < 0.001; NS no significant difference. HLD is hyphal length density.

**Table 2:** Results (*F*-value) of three-way factorial ANOVA on effects of elevated temperature (T), N addition (N) and arbuscular mycorrhizal fungi (AMF) on plant species composition and productivity.

Source of variance	Species diversity (*H*)	Species richness (R)	Species evenness (*E*)	Total aboveground biomass
T	12.80**	11.31**	3.476NS	85.23***
N	13.75**	3.21NS	2.68NS	92.87***
AMF	7.65*	2.05NS	6.48*	192.89***
T × N	3.22NS	5.77*	1.01NS	24.56***
T × AMF	5.03*	3.07NS	5.23*	11.75**
N × AMF	6.21*	6.55*	6.97*	46.91***
T × N × AMF	2.61NS	1.23NS	3.45NS	16.52***

*Significant difference at *P* < 0.05; **significant difference at *P* < 0.01; ***significant difference at *P* < 0.001; NS no significant difference.

**Table 3:** Results (*F*-value) of three-way factorial ANOVA on effects of elevated temperature (T), N addition (N) and arbuscular mycorrhizal fungi (AMF) on individual aboveground biomass and species composition (%) of total aboveground biomass.

Source of variance	Individual aboveground biomass					Species composition (%) of total aboveground biomass				
	*L. chinensis*	*S. viridis*	*L. davurica*	*C. virgata*	*S. corniculata*	*L. chinensis*	*S. viridis*	*L. davurica*	*C. virgata*	*S. corniculata*
T	89.655***	1.59NS	5.14*	1.33NS	37.69***	24.18***	3.55NS	1.12NS	2.01NS	1.13NS
N	15.48**	2.31NS	1.09NS	1.40NS	4.29*	0.05NS	6.54*	0.79NS	1.09NS	6.80*
AMF	30.06***	32.77***	2.48NS	0.43NS	41.94***	17.64***	53.82***	0.68NS	0.62NS	157.47***
T × N	23.31**	2.68NS	1.62NS	1.06NS	4.47*	0.26NS	0.56NS	1.05NS	1.06NS	0.33NS
T × AMF	10.73**	6.60*	0.95NS	1.04NS	41.38***	3.19NS	3.69NS	1.33NS	0.41NS	3.14NS
N × AMF	0.65NS	1.72NS	2.97NS	2.91NS	1.19NS	5.23*	0.01NS	1.19NS	0.38NS	7.67*
T × N × AMF	6.61*	1.47NS	2.13NS	1.37NS	9.59**	0.43NS	2.09NS	0.74NS	0.96NS	0.09NS

*Significant difference at *P* < 0.05; **significant difference at *P* < 0.01; ***significant difference at *P* < 0.001; NS no significant difference.
