# Supplementary material for: Response of AM fungi spore population to elevated temperature and nitrogen addition and their influence on the plant community composition and productivity
Source: Sci Rep. 2016 Apr 21;6:24749. doi: 10.1038/srep24749 (PMC4838856; doi:10.1038/srep24749)
Supplement: Supplementary Information [file srep24749-s1.doc]

**Supporting Information**

**Response of AM fungi to elevated temperature and nitrogen addition and their influence on the plant community composition and productivity**

Tao Zhang^1,3*^, Xue Yang^1^, Rui Guo^2^, Jixun Guo^1*^

^1^ Institute of Grassland Science, Northeast Normal University, Key Laboratory of Vegetation Ecology, Ministry of Education, Changchun 130024, China

^2^ Institute of Environment and Sustainable Development in Agriculture, Chinese Academy of Agricultural Sciences, Key Laboratory of Dryland Agriculture, Ministry of Agriculture, Beijing 100081, China

^3^ State Key Laboratory of Desert and Oasis Ecology, Xinjiang Institute of Ecology and Geography, Chinese Academy of Sciences, Urumqi 830011, China

*** Correspondence author:** Tao Zhang, Jixun Guo; tel:+86 431 85098937; fax: +86 431 85695065. e-mail: [zhangt946@nenu.edu.cn](mailto:zhangt946@nenu.edu.cn); [gjixun@nenu.edu.cn](mailto:gjixun@nenu.edu.cn).

**Table S1** List of AMF species within the experiment quadrats of experiment 1 at the beginning year and fourth year

| AMF species | Treatments  (At the beginning of first year) | | | | Treatments  (At the end of fourth year) | | | |
| --- | --- | --- | --- | --- | --- | --- | --- | --- |
|  | C | N | W | W+N | C | N | W | W+N |
| *Glomus aggregatum* | + | + | + | + | + | + | + | + |
| *G. claroideum* | + | + | + | + | + | + | + | + |
| *G. constrictum* |  |  |  |  |  |  |  | + |
| *G. clarum* |  |  | + |  |  |  | + |  |
| *G. constrictem* | + | + | + | + | + | + | + | + |
| *G. deserticola* | + | + | + | + | + | + | + | + |
| *G. etunicatum* | + | + | + | + | + |  | + | + |
| *G. fasciculatum* | + | + | + | + | + | + | + | + |
| *G. geosporum* |  |  |  |  |  |  | + |  |
| *G. intraradices* | + | + | + | + | + | + |  | + |
| *G. mosseae* | + | + | + | + | + | + | + | + |
| *G. rubisforum* | + | + | + | + | + | + |  | + |
| *G. versiforme* | + |  | + | + |  |  | + |  |
| *G. ambisporum* |  |  |  |  |  |  | + |  |
| *G.sp1* |  |  |  |  |  | + | + | + |
| *G.sp2* |  |  |  |  |  | + |  |  |
| *G.sp3* | + | + | + | + | + |  |  |  |
| *Claroideoglomus luteum* | + |  | + | + | + |  |  | + |
| *Acaulospora trappei* | + | + | + | + | + | + | + | + |
| *A. gerdemmnnii* | + | + | + | + | + | + | + | + |
| *A. rehmii* | + | + | + | + | + | + | + | + |
| *A. colossica* | + | + | + | + | + | + | + | + |
| *A.sp1* |  |  |  |  |  |  | + |  |
| *A.sp2* | + | + | + | + | + | + | + | + |
| *Entrophospora infrequens* | + | + | + | + | + |  | + |  |
| *Paraglomus occultum* | + | + | + | + | + | + | + | + |

“+” representAMF species were present in samples.

**Table S2** Mycorrhizal colonization in root system of each species in experiment 2

| Plang species | C | | N | | W | | W+N | |
| --- | --- | --- | --- | --- | --- | --- | --- | --- |
|  | NM | AM | NM | AM | NM | AM | NM | AM |
| *L. chinesis* | - | 66.7±5.6b | - | 55.4±4.8c | - | 83.2±5.8a | - | 65.6±6.2bc |
| *C. virgata* | - | 89.2±3.7a | - | 75.8±5.5b | - | 53.3±2.7c | - | 66.7±8.2b |
| *S. viridis* | - | 92.5±2.1a | - | 68.8±8.4b | - | 65.0±7.5b | - | 73.3±8.8b |
| *L. davurica* | - | 78.3±8.4a | - | 55.6±8.6b | - | 37.5±7.6c | - | 67.8±7.3ab |
| *S. corniculata* | - | - | - | - | - | - | - | - |

“-” represent no mycorrhizal colonization. Lowercase letters in each row represent significant difference among different treatments at 0.05 level.

**Table S3** Mycorrhizal benefits of these five species in different treatments.

| Treatments | Species | | | | |
| --- | --- | --- | --- | --- | --- |
|  | *L. chinensis* | *S. viridis* | *L. davurica* | *C. virgata* | *S. corniculata* |
| C | 240.6 | 258.5 | 25.4 | -83.4 | -34.0 |
| N | 51.1 | 47.4 | 186.8 | -1.6 | -25.5 |
| T | 366.0 | 374.4 | 57.9 | -13.7 | -65.1 |
| T+N | 743.4 | 429.9 | 43.2 | -32.3 | -78.6 |

C, control; N, N addition; T, elevated temperature; T+N, elevated temperature plus N addition. “-” represents minus.
